# Essential Oils: Chemistry and Pharmacological Activities—Part II

**DOI:** 10.3390/biomedicines12061185

**Published:** 2024-05-27

**Authors:** Damião Pergentino de Sousa, Francisco de Assis Oliveira, Daniel Dias Rufino Arcanjo, Diogo Vilar da Fonsêca, Allana Brunna S. Duarte, Celma de Oliveira Barbosa, Thomas Prates Ong, Timothy John Brocksom

**Affiliations:** 1 Laboratory of Pharmaceutical Chemistry, Federal University of Paraíba, João Pessoa 58051-900, Brazil; allanabrunna@gmail.com; 2 Medicinal Plants Research Center, Federal University of Piauí, Teresina 64049-550, Brazil; fassisol@ufpi.edu.br; 3 LAFMOL—Laboratory of Functional and Molecular Studies in Physiopharmacology, Department of Biophysics and Physiology, Federal University of Piaui, Teresina 64049-550, Brazil; daniel.arcanjo@ufpi.edu.br (D.D.R.A.); celmaoliver@yahoo.com.br (C.d.O.B.); 4 Collegiate of Medicine, Federal University of São Francisco Valley, Bahia 48607-190, Brazil; divilar@hotmail.com; 5 Department of Food Science and Nutrition, School of Pharmaceutical Sciences, University of São Paulo (USP), São Paulo 05508-000, Brazil; tong@usp.br; 6 Food Research Center (FoRC), University of São Paulo, São Paulo 05508-000, Brazil; 7 Department of Chemistry, Federal University of São Carlos, São Carlos 13565-905, Brazil; brocksom@terra.com.br

**Keywords:** essential oil, cardiovascular, antidiabetes, antiulcer, cancer chemopreventive, organic synthesis, natural products, metabolites, medicinal plants, volatiles

## Abstract

The importance of essential oils and their components in the industrial sector is attributed to their chemical characteristics and their application in the development of products in the areas of cosmetology, food, and pharmaceuticals. However, the pharmacological properties of this class of natural products have been extensively investigated and indicate their applicability for obtaining new drugs. Therefore, this review discusses the use of these oils as starting materials to synthesize more complex molecules and products with greater commercial value and clinic potential. Furthermore, the antiulcer, cardiovascular, and antidiabetic mechanisms of action are discussed. The main mechanistic aspects of the chemopreventive properties of oils against cancer are also presented. The data highlight essential oils and their derivatives as a strategic chemical group in the search for effective therapeutic agents against various diseases.

## 1. The Transformation of Readily Available Essential Oil Constituents into High-Value Products

Essential oils have been used by humanity for thousands of years, basically due to their very pleasant aromas, flavors, and relevant pharmacological properties. This has led to the creation of the flavor and fragrance industry and a significant participation in the pharmaceutical industry. The major modification observed over the last two centuries is the change from the direct utilization of the essential oils and their major constituents to new chemical products obtained by their synthetic transformations. The principal essential oils usually present a major component, associated with a characteristic aroma, and they are potentially a great starting material for more complex compounds of highly increased commercial value. In this review, we describe and discuss some major essential oils, their principal components, and their synthetic transformations into more highly valued products [1,2]. Clearly, essential oils are an important part of the renewable biomass already in use.

Essential oils, as obtained from nature by classical methods, are generally composed of very complex mixtures of terpenoids and phenylpropanoids, both groups being of scientific and commercial interest. The major constituents are monoterpenes with 10 carbon atoms, in various carbon skeleton arrangements, with none or few functional groups, but usually available in both enantiomeric forms. Organic chemists describe these compounds as members of the chiral pool, an extremely important gift from nature that allows us to synthesize much more complex molecules, both of academic and industrial interest, in their pure enantiomeric forms [3]. As is well accepted, odor, flavor, and pharmacological properties are all directly related to their enantiomeric purity. 

**Figure 1:**
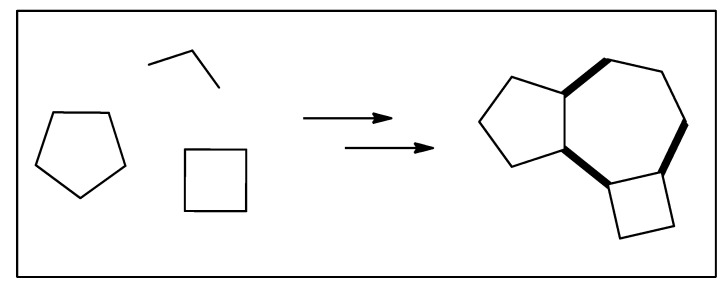
Organic synthesis, the constructive process.

At this point, we should mention organic synthesis as being the science that allows the transformation of these relatively simple essential oil constituents into the desired finished products. A parallel situation exists in nature where simple molecules are transformed into much more complex molecules, and where nature is the supreme artist in this activity. Synthetic transformation is a sequence of constructive reactions in which the starting material molecule is structurally modified in the direction of the desired and proposed final molecules [4] (Figure 1).

This process is interactive, involving planning known as retrosynthetic analysis and then constructive execution of the strategy, with consequent modifications both of the plan and the execution as determined by the ongoing experimental results obtained [5].

The latest experimental revolution is the introduction of a new enabling technology for synthetic execution. The traditional equipment set-up is the batch reactor made of glass or metal, in which a defined and limited quantity of starting materials are transformed into products. We are now using the continuous flow technology [6,7] in which the starting materials are pressurized into a continuous flow and then pass through the requisite reactors, and other required equipment, leading then to the collector. This technology can be run even on a 24 h per day routine, and obviously multiplied up with parallel systems. This technology is being transplanted from the research environment into the pharmaceutical industry with increasing intensity and success.

We shall focus on Brazilian essential oils, which represent a major world market but are also similar to those of other major third world producers; that is, mainly exportation of the crude essential oils but relatively little local synthetic exploration.

**Figure 2:**
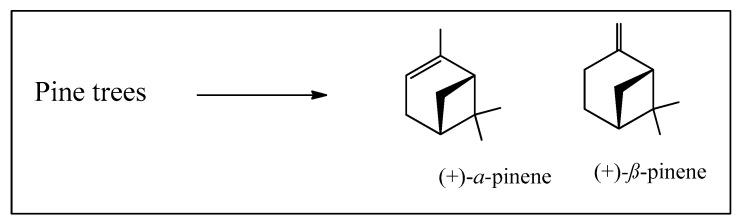
Pine trees and the pinenes.

We will start with pine trees and their essential oils, historically known as turpentine, now substituted by petrochemically derived solvents in the paint industry. Pine trees are excellent examples of industrial excellence, as they furnish wood and thus paper, and as a secondary product, the essential oil is obtained from other parts of the tree. It is difficult to provide accurate and reliable amounts of the different essential oils being produced around the world. However, we will estimate turpentine oil production as being of the order of 400,000 tons/year worldwide. Certainly, the other major essential oils are being produced on much more modest scales. *Pinus elliottii* Engelm. (slash pine) has been successfully adapted to many parts of southern Brazil and, together with *Pinus taeda* L., is our main source of pinenes (Figure 2).

These essential oils are mainly composed of *α*- and *β*-pinenes, present in varying percentages, and one of the enantiomeric forms depending upon the species (Figure 2). What can we do with them apart from using their excellent dissolving properties [8,9]? We have decided to carefully select examples of industrial transformations, which involve large-scale production while using relatively simple chemical processes. However, we will not detail further the major industrial operation which is the production of diverse polymers, including “natural” rubbers.

The major transformation of the pinenes is in the production of *α*-terpineol for its fragrance qualities and antimicrobial activity, already executed under continuous flow conditions [10]. A personal affirmation is that a natural chemical is identical to its synthetic version, only differing in their respective impurities, availabilities, and price.

**Figure 3:**
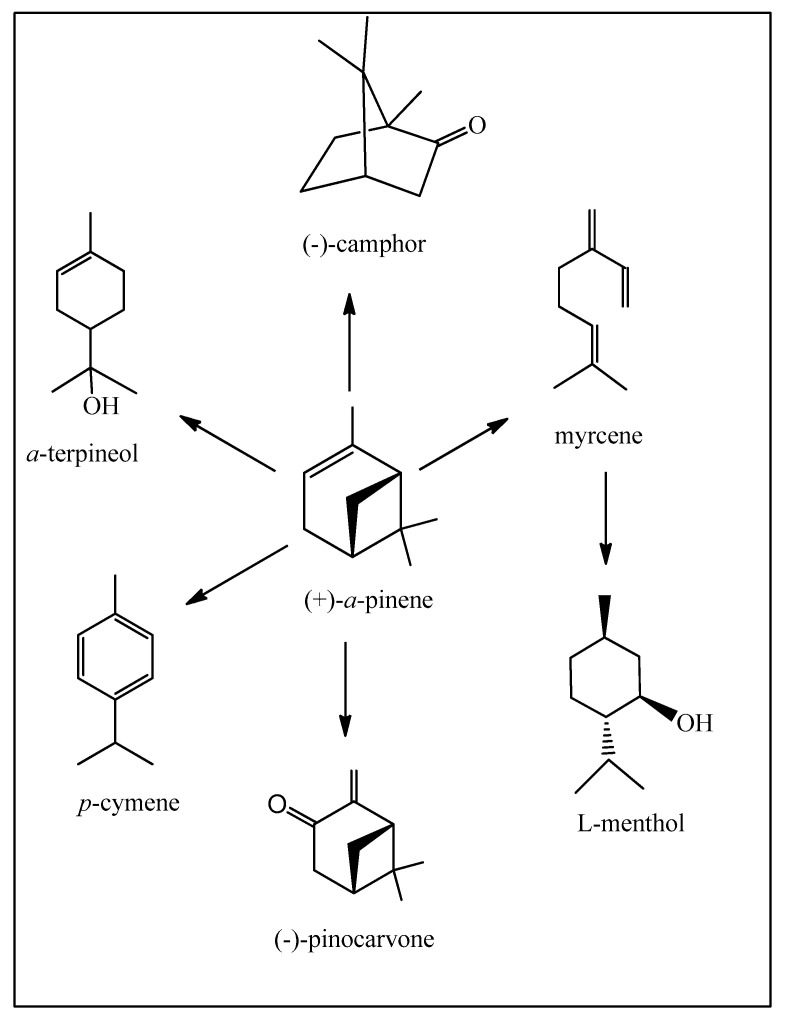
The pinenes to *α*-terpineol, camphor, *p*-cymene, pinocarvone, and myrcene (for L-menthol).

As a general rule we will not detail the synthetic transformations or specify the individual reactions performed but simply present the overall process from starting materials to products. The pinenes are also used to produce camphor, *para*-cymene, and myrcene (for transformation into L-menthol). These compounds have important fragrance and pharmacological properties. *para*-Cymene can be envisaged as a green solvent which can substitute the petrochemically derived solvents benzene, toluene, and the xylenes (Figure 3). The photo-oxidation of *α*-pinene to pinocarvone can be executed under continuous flow conditions [11,12].

**Figure 4:**
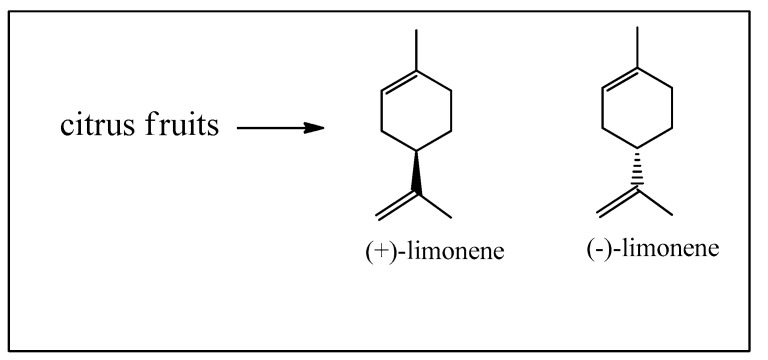
Citrus fruits and the limonenes.

The second major starting materials are the enantiomeric limonenes, produced from citrus fruit essential oils obtained from the peel, as a secondary product to the immense citrus fruit juice market (Figure 4) [13].

**Figure 5:**
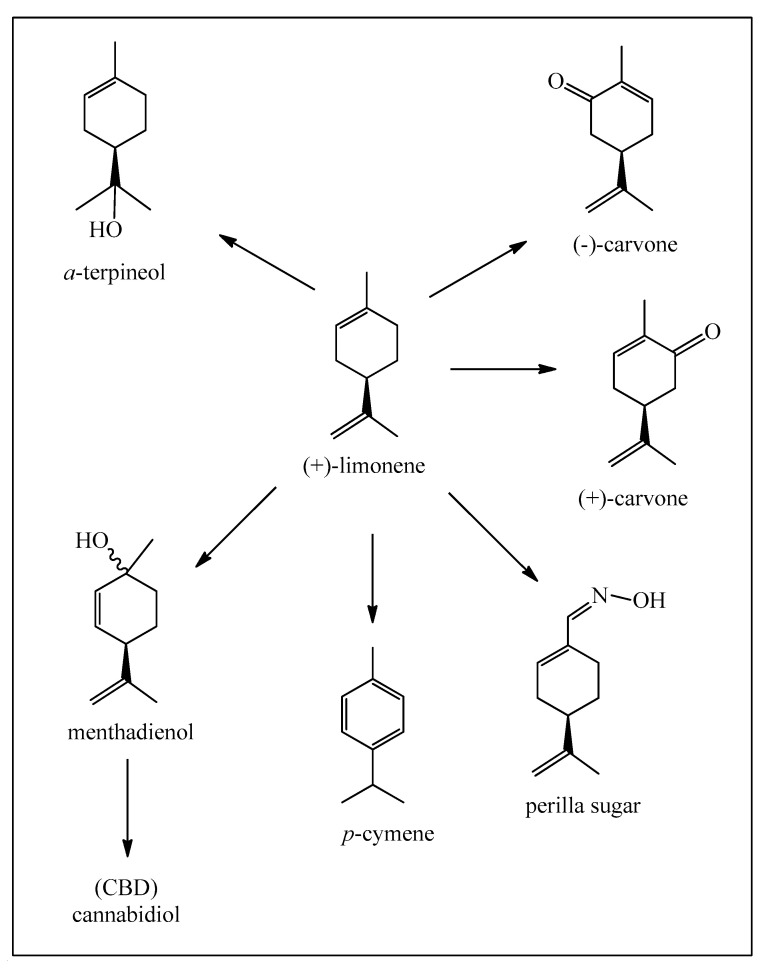
The limonenes to α-terpineol, *para*-cymene, perilla sugar, and the carvones; menthadienol and CBD.

**Figure 6:**
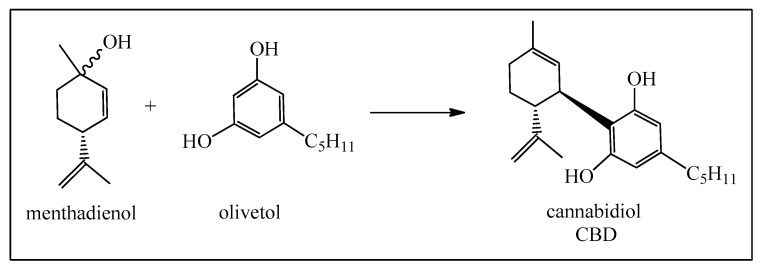
Menthadienols plus olivetol lead to cannabidiol.

These compounds have a much simpler molecular structure than the pinenes but are also utilized in the production of *α*-terpineol. Besides important polymer production, limonenes are also used for the synthesis of *para*-cymene and enantiomeric carvones. Limonenes can be selectively oxidized at the isolated methyl group, leading finally to the oxime of perillaldehyde, usually known as perilla sugar, an excellent sweetener. Limonene can also be photo-oxidized to the *p*-menthadienol epimers (Figure 5) [14], and then directly transformed into cannabidiol (CBD) as shown in Figure 6 [15,16]. At this point, we should point out that the legalized and highly recommended cannabidiol (CBD) can be synthesized easily from limonene and also from *α*-pinene, citral, and isopulegol. A great advantage is that synthetic cannabinoids do not contain the psychoactive tetrahydrocannabinol (Δ^9^-THC), as opposed to mixtures isolated from *Cannabis sativa* L. which do and therefore are subjected to very strict legal controls.

**Figure 7:**
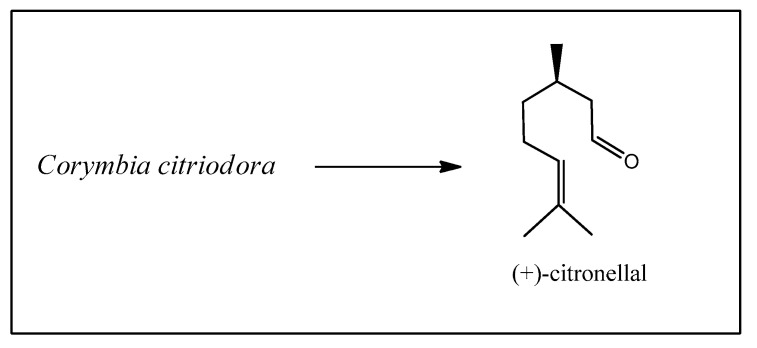
*Corymbia citriodora* and citronellal.

The next starting material is citronellal, obtained from the essential oil of *Corymbia citriodora* (Hook.) K.D. Hill & L.A.S. Johnson, a *Eucalyptus* species widely grown in Brazil for paper and pulp production (Figure 7) [17].

**Figure 8:**
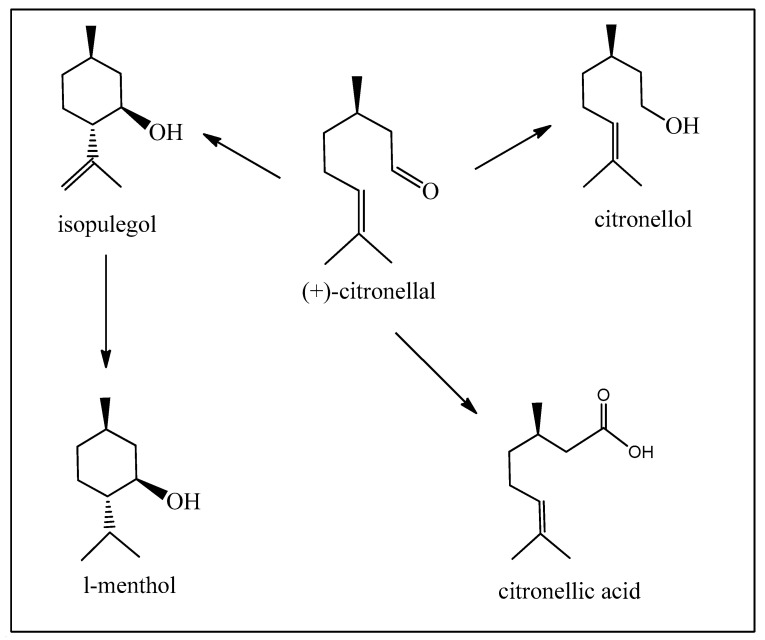
Citronellal derivatives; L-menthol from citronellal by way of isopulegol.

Citronellal should be the best starting material for obtaining L-menthol, a compound with desirable flavor and fragrance with local anesthetic and cooling properties. However, L-menthol is presently obtained from *Mentha piperita* L. essential oil, and the major part is produced by other synthetic procedures on a reasonably large industrial scale (Figure 8). The most important and acclaimed synthesis starts from the pinenes, which was developed by the company Takasago, and is responsible for the major quantities available worldwide (see Figure 3) [18,19,20]. This is basically due to the greater availability of pinenes.

**Figure 9:**
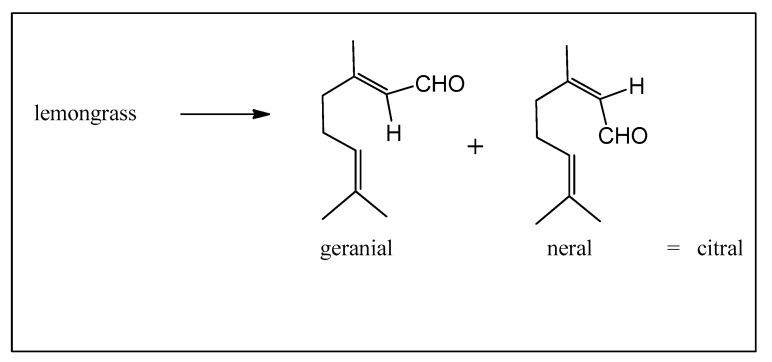
Citral, geranial, and neral from lemongrass.

Citral, a variable mixture of geranial and neral, is available in several essential oils including lemongrass (*Cymbopogon genus*), widely grown in Brazil as a home remedy (Figure 9).

**Figure 10:**
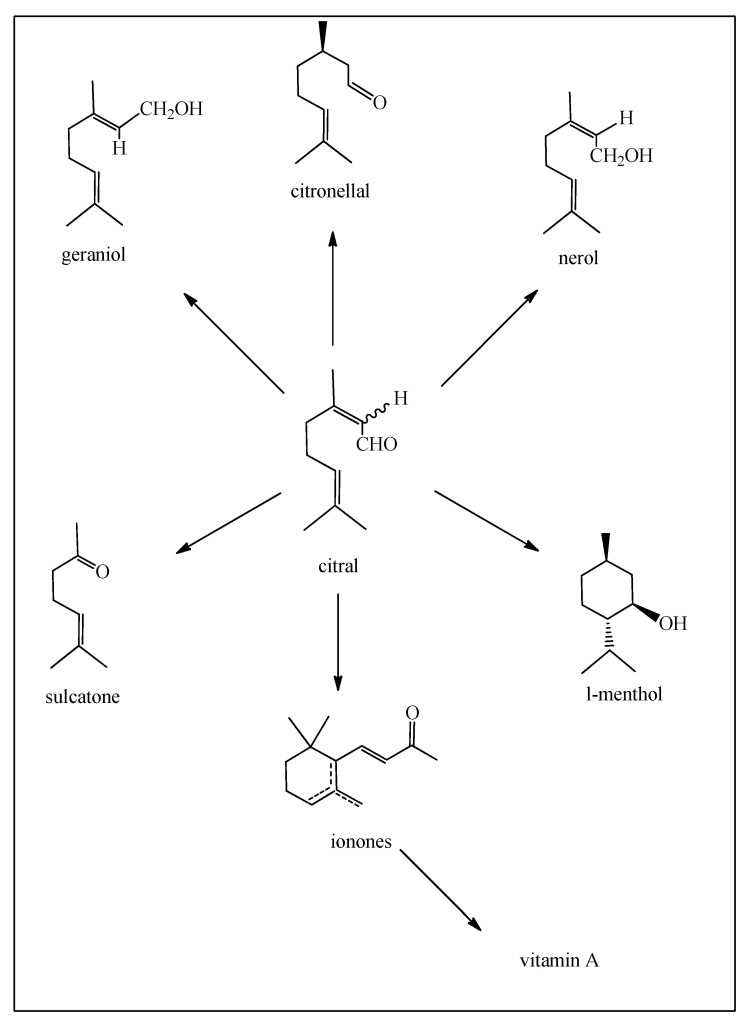
Citral-derived products.

Citral can be used for the production of L-menthol [18,21], pseudo-ionone [22], the ionones, and then on to vitamin A (Figure 10), and also for certain certified cannabinoids.

**Figure 11:**
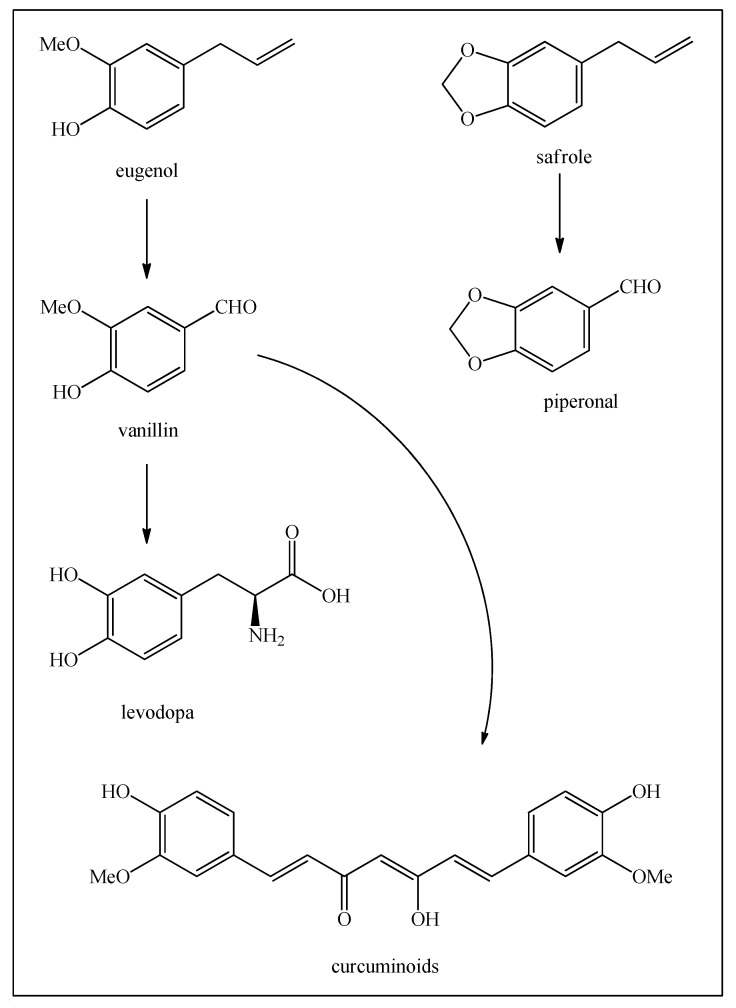
Eugenol to vanillin, levodopa, and curcuminoids.

Of the phenylpropanoids, eugenol (found in many herbs, including cloves and cinnamon) is certainly the most useful at the moment, being transformed into vanillin, and is also available on a much larger scale from sugarcane lignins. Vanillin is then the starting material in the syntheses of levodopa and the curcuminoids (Figure 11) [23].

In a different synthetic strategy, essential oil constituents can be envisaged as starting materials in chemoenzymatic syntheses [24,25,26,27], also known as biocatalysis, in which (modified) enzymes are called into play. This is especially interesting when organic syntheses are shown to be less effective. The reason for this is usually the lack of selectivity in classical chemical reactions, with the formation of several products in lower yields, and demanding extensive mixture separations. The chemoenzymatic alternative, when possible, affords much higher selectivity and thus higher yields and easier product purification. At the present moment, much academic activity is clearly demonstrating the enormous potential of this alternative. We can imagine, in the very near future, syntheses involving one or more chemoenzymatic reactions together with more classical chemical reactions.

**Figure 12:**
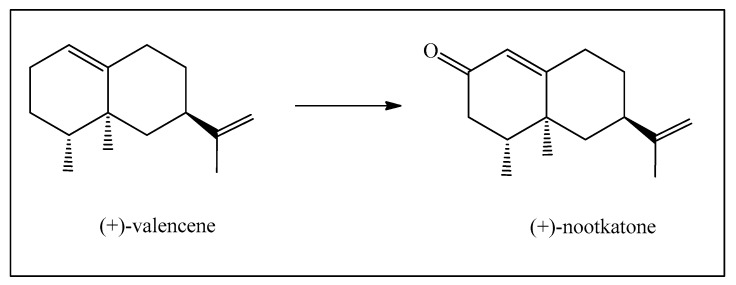
Valencene to nootkatone.

An excellent example is the transformation of valencene, isolated from certain citric fruit essential oils, into the fragrance nootkatone with a very marked grapefruit-like aroma. Although valencene obtained from the essential oil is not very pure and is now becoming relatively expensive, nootkatone is a highly prized product (Figure 12) [28].

We should emphasize that all these readily available starting materials have been extensively used in elaborate syntheses to obtain highly complex molecules of more limited commercial interest. Here, we have concentrated on simpler synthetic procedures leading to high volume and commercially relevant products. The novelties reside in the new technological processes, principally continuous flow instead of batches, and chemoenzymatic and biocatalytic reactions substituting more conventional reagents.

## 2. Mechanisms of Pharmacological Action of Essential Oils

### 2.1. Mechanisms of Antiulcer Action of Essential Oils

Peptic ulcer disease (PUD) occurs in the stomach (gastric ulcer) and the first portion of the small intestine (duodenal ulcer). Signs and symptoms can include abdominal pain, upper gastrointestinal bleeding, gastric outlet obstruction, and perforation [29]. Among all complications, peptic ulcer bleeding is one of the most common clinical diseases [30] and its morbidity has not decreased [31]. The wounds caused by PUD can penetrate the gastric mucosa and reach the muscular coat, subsequently forming a cavity characterized by different stages of necrosis, neutrophil infiltration, blood flow reduction, increased oxidative stress, and acute and chronic inflammation [32,33].

PUD is a source of significant morbidity and mortality worldwide, with much of the burden in low-income and middle-income countries [34]. The prevalence of PUD has been estimated at around 5–10% [35] and it affects 4 million people worldwide annually [36]. Disequilibrium between defensive factors (bicarbonate production, mucus secretion, prostaglandins (PGs), nitric oxide (NO), and antioxidants) and aggressive factors (oxidative stress and free radicals, acid and pepsin, fall in the gastric blood flow, *H. pylori* infection, and non-steroidal anti-inflammatory drugs (NSAIDs)) in the gastric mucosa is reported to be a fundamental mechanism involved in the pathogenesis of disease peptic ulcer disease [30]

The decrease in the incidence of peptic ulceration in the last 30 years is partly due to the substantial progress in the pharmacological management of dyspeptic symptoms as well as to a significant understanding of its pathophysiology [37]. Management of PUD entails reducing the production of gastric acids and includes antiacids (sodium bicarbonate, aluminum hydroxide), H2 receptor antagonists (cimetidine, ranitidine, famotidine), proton pump inhibitors (omeprazole, lansoprazole, pantoprazole), and cytoprotective agents (sucralfate, bismuth or prostaglandin analogs). In the case of a gastric ulcer associated with *H. pylori*, treatment consists of a combination of antibiotics, such as amoxicillin or clarithromycin, and acid secretion inhibitors [38].

Despite good therapeutic efficacy, these agents often cause severe side effects, such as osteoporosis, gynecomastia, hypoacidity, hypergastrinemia, iron and vitamin B12 deficiencies, thrombocytopenia, and cardiovascular disease risks, that limit their clinical use [39,40,41,42,43,44].

For these reasons, research for the development of new gastroprotective agents that are safer for long-term use has attracted interest. Given this, natural products (medicinal/herbal plants and extracts) are considered attractive sources for potential new drugs and have shown promising results for the treatment of gastric ulcers [45,46,47]. The efficacy of herbal medicines is comparable or superior to synthetic drugs with fewer adverse effects and they have been proven as one of the best strategies for the disease management of ulcers [48,49].

#### 2.1.1. Antioxidant Activity

*Hyptis martiusii* Benth. (Lamiaceae) is an aromatic plant found in northeastern Brazil. The leaves of this plant are used against diseases of the stomach and intestine [50]. Caldas et al. [51] characterized the mechanisms of action involved in the gastroprotection of the essential oil of *H. martiusii* (EOHM) using an ethanol-induced gastric ulcer model in rats. Treatment with EOHM (400 mg/kg) decreased the rate of lipid peroxidation by significantly diminishing the production of malondialdehyde and reversing the reduction in levels of sulfhydryl groups in the mucosa in ethanol-induced gastric mucosal lesions in rats. In an in vitro study of radical scavenging activity (DPPH assay), the free radical scavenging ability of the EOHM showing that the EOHM exhibited no significant relative ability to promote the capture of the DPPH radical at any of the concentrations tested. Similarly, 1,8-cineole, a major component of the essential oil, was unable to promote the capture of DPPH.

In a new study with 1,8-cineole, also called eucalyptol, Caldas et al. [52] showed that oral administration of this monoterpene (100 mg/kg) was able to revert the reduction of the levels of sulfhydryl groups in the gastric mucosa by 62%, restoring the antioxidant system to base levels in the gastric ulcer model induced by ethanol in Wistar rats. The monoterpene decreased the rate of lipid peroxidation and myeloperoxidase activity (MPO) by 55.3% and 59.4%, respectively, by diminishing the production of malondialdehyde by ethanol when compared to the injured control group. Acute administration of ethanol to rodents produces gastric mucosal damage that involves intracellular oxidative stress, intracellular thiol groups, and microcirculation disturbances [53]. These results suggest the involvement of an antioxidant mechanism in the gastroprotective activity of 1,8-cineole and that compound action is related to the cytoprotection effect of the essential oil of *H. martiusii*.

*Syzygium aromaticum* L., known as clove, is a dried flower bud belonging to the Myrtaceae, rich in volatile compounds and antioxidants such as eugenol and α-humulene. Clove essential oil (CEO) is traditionally used in many disorders. The literature evidenced several remarkable properties, such as treatment for tooth infections and toothache [54] and antiangiogenic [55], antioxidant [56], and anti-inflammatory activities [57]. Recently, Hobani et al. [58] reported that eugenol (5 and 10 mg/kg, p.o.) exhibited a reduction of gastric damage by ethanol-induced gastric ulceration. The extent of inhibition was 83.55 and 41.06%, respectively. In addition, eugenol protected the NP-SH and GSH levels, and it reduced the gastric tissue MDA level. Alcohol causes depletion of the gastric wall mucosal barrier via ROS formation and an increase in permeability [59]. In turn, ROS generation can cause oxidative stress, with the main source of ROS being infiltrating inflammatory cells, such as neutrophils that react with lipids to produce lipid peroxidation [60]. This study demonstrates that eugenol has gastroprotective properties against ethanol-induced ulcers, probably via improvement of cellular antioxidant defense. These results are in line with the works carried out by Barboza et al. [61] and Jung et al. [62] who demonstrated that eugenol exerts an action on oxidative stress and exhibits antioxidant activity and a protective effect against gastric damage, suggesting that eugenol has therapeutic potential for the treatment of inflammatory conditions such as gastritis.

#### 2.1.2. Mucosal Proliferation

Pogostemonis Herba is a known traditional Chinese medicinal herb in Southeast Asia. It originates from the dried aerial part of *Pogostemon cablin* (Blanco) Benth. (Labiatae). Clinically, Pogostemonis Herba has been used by traditional Chinese physicians to treat a large number of medical conditions, for example, common cold, diarrhea, and *H. pylori*-related gastritis [63]. According to the literature, among the majority components of *P. cablin* (Blanco) Benth. essential oil (Lamiaceae) are patchouli alcohol, *β*-patchoulene, and pogostone, which have anti-inflammatory and gastroprotective activities [64,65,66].

Patchouli alcohol is a tricyclic sesquiterpene and also the major active ingredient from the essential oil of Pogostemonis Herba. This terpene has exhibited diverse pharmacological activities, for instance, enhancing cognition in mice with memory impairment induced by scopolamine and anti-inflammatory and anti-influenza virus activities in vitro and vivo [67]. A study developed by Zheng et al. [68] investigated the antiulcerogenic properties of patchouli alcohol (PA) against various experimental gastrointestinal ulcerations in rats. Animal groups pretreated with PA (10, 20, and 40 mg/kg mg/kg, p.o.) exhibited a dose-dependent reduction of gastric damage by ethanol-induced gastric ulceration. The extent of inhibition for the doses employed was 49.20%, 63.89%, and 71.85%, respectively. The experimental model of ethanol-induced gastric ulcers is often employed to screen antiulcer compounds [64]. Ethanol damages the gastric mucosa by diverse routes, including the generation of oxygen-derived free radicals, lipid peroxidation, depletion of mucus, the reduction of antioxidant defense, and decreased prostaglandin level [69].

To verify the participation of mucus secretion in the gastroprotective effect of PA, the pylorus ligation-induced ulcer rat model was used. This model undergoes hypersecretion which leads to the accumulation of gastric acid in the stomach lumen and promotes the generation of oxidative free radicals [70], resulting in autodigestion of gastric mucosa and breakdown of the mucosal barrier [71]. The mucus layer is the primary defense of the gastric mucosa. It acts as a physical barrier against the aggressive effect of gastric juice [72]. Increased mucus secretion can increase the buffering of acids in gastric juice and can reduce mucosal damage mediated by oxygen free radicals [73]. Agents that increase the secretion of mucus are effective in preventing the ulcers induced by this method [74]. The authors showed that the amount of adhered gastric mucus was augmented (*p* < 0.05) by pretreatment with PA compared to the control group. In line with this view, it could be said that PA protects the gastric mucosa through enhanced mucus secretion and potentially plays an important role in gastric mucosal protection.

#### 2.1.3. H^+^/K^+^ ATPase Activity

*Cymbopogon citratus* (DC) Stapf, belonging to the family Poaceae, popularly known as lemongrass, is an aromatic plant used in Brazilian popular medicine for the treatment of gastric and nervous disorders [75]. The essential oil from *C. citratus* (EOCC) is mostly composed of the monoterpenes citral, nerol, and geraniol [76]. Venzon et al. [77] showed that EOCC, citral, and geraniol at doses of 1–100 mg/kg (p.o.) exerted marked protection of the gastric mucosa in an acute ethanol-induced ulcer model. In addition, it was demonstrated that the essential oil of *C. citratus* and citral at 100 μg/mL were able to inhibit, in vitro, the activity of the H^+^/K^+^ ATPase by 28.26% and 44.36%, respectively. However, the geraniol did not inhibit this enzyme. The gastric H^+^/K^+^ ATPase, a member of the P2-type ATPase family, found in gastric parietal cells, is known to transport H^+^ against a concentration gradient, leading to acid secretion [78]. It has been well-established in the literature that effective proton–potassium ATPase inhibitors (PPIs) are potential antiulcerative agents since they interfere with the cascade of events of gastric ulcerations [79]. PPIs play a crucial role in the management of gastroesophageal reflux disease, Barrett’s esophagus, and in the treatment of *Helicobacter pylori* infection [80]. Based on the findings, it is possible to infer that the inhibition of the effect of the proton pump exerted by EOCC and citral may be related to their gastroprotective effect and that is not an essential mechanism involved in the gastric healing effects promoted by geraniol.

#### 2.1.4. Nitric Oxide

Nitric oxide (NO), a potent vasodilator, appears to be a major regulator of blood flow and gastric microcirculation [81]. NO has been shown to protect against ethanol-induced gastric lesions, whereas inhibition of NO synthesis (NOS) has been demonstrated to increase the susceptibility of the stomach to ethanol injury [82].

*Croton rhamnifolioides Pax* & Hoffm. (Euphorbiaceae) is an aromatic plant species used in folk medicine to treat inflammation and ulcers [83,84]. A previous study identified 57 compounds in the essential oil of *C. rhamnifolioides*, whose major constituents were sesquicineole, *α*-phellandrene, 1,8-cineole, and (*E*)-caryophyllene [85]. Martins et al. [86] investigated the anti-inflammatory activity of the essential oil from *C. rhamnifolioides* leaves (OEFC) and 1,8-cineole, its major constituent. OEFC (25 mg/kg) and 1,8-cineole (10.33 mg/kg) reduced edema in several models of inflammation (histamine, arachidonic acid, carrageenan, croton oil, 1% dextran, and granuloma), indicating regulatory action on the release of inflammatory mediators. Intending to investigate the gastroprotective properties of this plant, Vidal et al. [87] evaluated *C. rhamnifolioides* essential oil (OECC) using the absolute ethanol, acidified ethanol, or indomethacin gastric lesion model in mice. Animals that were pretreated orally with OECC (200 mg/kg) showed a significant reduction in lesions. Administration of L-arginine (600 mg/kg, i.p.), a substrate for NOS, also reduced the lesion area, and the pretreatment of L-NAME (20 mg/kg, i.p.), an inhibitor of nitric oxide synthase, reversed both protective effects of L-arginine and OECC, suggesting the likely participation of nitric oxide in the gastroprotective activity of OECC. In agreement with this study, Lima-Accioly et al. [88] demonstrated that the gastroprotective effect of an essential oil obtained from *Croton nepetaefolius* is associated with the same mechanism.

Nitric oxide (NO) appears to be a key mediator of gastrointestinal mucosal defense mechanisms, such as gastric blood flow and gastric microcirculation [89]. Nitric oxide synthase (NOS)-derived NO causes vascular dilation by stimulating soluble guanylyl cyclase and increasing cGMP in the smooth muscle cells. Therefore, nitric oxide plays an important role in the gastric mucosa against ethanol-induced gastric lesions, and conversely, inhibition of NO synthesis increases the susceptibility of the stomach to ethanol injury [90]. In conclusion, when OECC is administered orally it exerts its gastroprotective activity by mechanisms that involve at least the participation of nitric oxide. 1,8-Cineole, the major component in OECC (18.32%), may be one of the components responsible for the observed effect [52].

#### 2.1.5. Prostaglandin E2 Levels

*Citrus* belongs to the family Rutaceae and is one of the main fruit tree crops grown throughout the world. *Citrus* essential oil is an important biologically active product from citrus peel. The citrus EO is composed of tens to hundreds of various compounds [91]. *Citrus lemon* Burm. f. (Rutaceae) is the third most important cultivated citrus species with a production of 7.3 million tons around the world annually [92]. The phytochemical analysis of *Citrus lemon* essential oil (CLEO) showed that its main compounds are two monoterpenes, limonene, and *β*-pinene [93].

Limonene, a monocyclic monoterpene, is a major constituent in several citrus oils (orange, lemon, lime, and grapefruit). It is effective in relieving heartburn and gastroesophageal reflux disorder. In animal studies, this monoterpene has also demonstrated chemoprotective activity for several types of cancer [94]. *β*-Pinene is a bicyclic hydrocarbon found in many essential oils from plants. This monoterpene has antimicrobial, anti-inflammatory, gastroprotective, and cytoprotective activities [95].

For establishing the gastroprotective action mechanism of CLEO and its main compounds, limonene and pinene, Rozza et al. [96] evaluated whether the gastroprotective effect from CLEO is due to its main compound limonene or the synergic activity of all components of this oil. To this end, three experimental models were used to investigate the bioactivity of the products: gastric ulcers induced by ethanol and indomethacin and their anti-*Helicobacter pylori* activity in vitro. The results indicate bioactive CLEO (250 mg/kg) and limonene (177 mg/kg) in gastroprotection (*p* < 0.05) in ethanol- and indomethacin-induced gastric ulcer models, while *β*-pinene (33 mg/kg) did not exert effective gastroprotection (53.26% and 37%, respectively). 

Indomethacin (IND) induces gastric mucosal damage by ROS through a significant increase in membrane lipid peroxidation [97]. In addition, IND blocks the gastroprotective effects of prostaglandin E2 (PGE2) that augments mucus and bicarbonate secretions as well as gastric blood supply [98]. Thus, the involvement of PGE2 in the gastroprotective effect of CLEO and limonene was evaluated using the indomethacin-induced ulcer model. The results showed that the gastroprotective effect of CLEO (250 mg/kg, p.o.) includes the maintenance of PGE2 levels with indomethacin administration (30 mg/kg). Meanwhile, in the limonene group, the results indicate that the gastroprotective effect against indomethacin is not related to PGE2 level maintenance. Thus, data analysis shows that maintenance of PGE2 levels is observed in the gastroprotective mechanism of CLEO, but it was not suggested in the limonene mechanism. 

Patchouli, known as *Pogostemon cablin* Benth. and belonging to the family Lamiaceae, is an important aromatic plant from Southeast Asia. Various bioactive compounds have been identified in patchouli and, among the compounds, pogostone is of great importance [99,100]. An earlier study by Chen et al. [101] reported the strong potential of pogostone in an indomethacin-induced gastric ulcer model in rats. Pogostone (10, 20, and 40 mg/kg) demonstrated inhibitory activity with the highest inhibition rate at a dose of 40 mg/kg (82.2%). Furthermore, a lower but still significant level of gastroprotection was obtained with 20 and 10 mg/kg pogostone resulting in an average gastric ulcer inhibitory rate of 56.6% and 47.5%, respectively. Pogostone (10, 20, and 40 mg/kg) also promoted the increment of gastric mucosal PGE2 levels (58.6, 64.9, and 67.6 ng/g) in a dose-related manner and significantly increased both COX-1 and COX-2 expressions. Additionally, pogostone (10, 20, and 40 mg/kg) significantly augmented endogenous SOD, GSH, and CAT activities, diminishing the constituent MDA level in the gastric mucosa in a dose-dependent manner. From their results, it was concluded that pogostone displayed gastroprotective action by enhancing gastric mucosal defensive factors which is rooted in the modulation of PGE2 levels and enhancement of the cellular antioxidant mechanism. Therefore, it could be a good therapeutic agent for the treatment of gastric ulcers.

#### 2.1.6. Reduction of Bacterial Colonization: *Helicobacter pylori*

Geraniol is an acyclic isoprenoid monoterpene found in essential oils of several aromatic plants, such as *Cinnamomum tenuipilum* Kosterm (Lauraceae) and *Cymbopogon citratu*s (DC) Stapf [77]. Furthermore, this monoterpene has been shown to exhibit important pharmacological properties, including antioxidant, anti-inflammatory, antimicrobial, and antitumor activities [102].

Recently, Bhattamisra et al. [103] investigated the antiulcer and anti-*Helicobacter pylori* activity of geraniol in an experimental chronic gastric ulcer model (acetic acid ulcers induced by submucosal injection of acetic acid). Treatment with geraniol at doses of 15 and 30 mg/kg (i.d.) resulted in fewer dilated blood vessels and hemorrhagic streaks on the gastric mucosal surface, producing a significant reduction in the ulcer index. The results showed that geraniol attenuated (*p* < 0.05) the extent of damage in the stomach induced by acetic acid by 42% and 52%, respectively.

In the rapid urease test (RUT), geraniol (15 and 30 mg/kg) showed a 17% and 33% reduction in *H. pylori*-positive antral samples, respectively. These results were supported by the histopathological data. Treatment with geraniol (30 mg/kg) reduced inflammation (*p* < 0.05) with a reduction in lesion scores and decreased bacterial load in the gastric mucosa in comparison to ulcer control in the *H. pylori* group. The RUT is an indirect test of the presence of *H. pylori* based on the presence of urease in the gastric mucosa [104]. The sensitivity of the RUT is high and has been reported to vary between approximately 80% and 100% with specificity between 97% and 99% [105]. Based on these findings, Bergonzelli et al. [106] determined that geraniol had a minimal inhibitory concentration (MIC) of 2 mg/L and inhibited 92% of *H. pylori* growth. 

The antiulcer and anti-*H. pylori* actions of geraniol may involve direct gastroprotective effects, as well as an antibacterial action against *H. pylori*. It is important to highlight the report of the great healing and gastroprotective activity of several monoterpenes. However, they do not have anti-*H. pylori* action (in vitro). Therefore, geraniol has potential use in the treatment of peptic ulcers associated with *H. pylori.*

#### 2.1.7. Inflammation: Role of Proinflammatory Cytokines

The acetic-acid-induced gastrointestinal ulcer model is similar to human ulcers in terms of location, severity, and chronicity. This model has proven suitable for investigating the effect of treatment on the healing process of chronic gastrointestinal ulcers [107] and it has been used to screen antisecretory and cytoprotective drugs [108]. Gastric ulcer occurs due to changes in multiple factors including prostaglandins, growth factor, nitric oxide, cytokine, microcirculation, and mucus adhesion [109]. After a gastric ulcer is induced by acetic acid, gastric inflammation increases interaction between leukocytes and endothelial cells characterized by the migration of macrophages in the ulcer area. The migrated macrophages then release proinflammatory cytokines such as TNF-*α* and interleukin-1*β* (IL-1*β*) [110,111]. TNF-*α* and IL-1*β* are considered parameters of the systemic inflammatory reaction and represent two important factors in the pathogenesis of gastric ulcers, contributing to many forms of gastric mucosal damage [112,113].

*Gallesia integrifolia* (Spreng.) Harms (Phytolaccaceae), a tree popularly known as the “garlic plant”, is a native and endemic plant of Brazil [114]. In traditional medicine, the bark of this species is utilized to prepare teas for treating ulcers, cough, flu, pneumonia, vermin, gonorrhea, prostate tumors, and rheumatism [115,116].

Arunachalama et al. [117] evaluated the gastric antiulcer action of the essential oil of the inner stem bark of *G. integrifolia* (EOGI). In this work, the authors described the protective effect of oral treatment of EOGI on gastric lesions induced by several necrotizing agents and found that EOGI (5, 20, or 80 mg/kg, p.o.) significantly reduced the levels of TNF-*α* and IL-1*β* in mice subjected to gastric ulcer induced by 99.5% acetic acid (20 mL). These results suggest that the ulcer healing effect of EOGI on gastric mucosal injury is probably related to a decrease in the TNF-*α* and IL-1 beta levels in inflammatory tissue. 

Chen et al. [66] evaluated the antiulcerogenic potential of pogostone using ethanol-induced gastric ulcers in rats as an experimental model. Pogostone (10, 20, and 40 mg/kg) exhibited a dose-dependent protective effect against ethanol gastric lesions (44.87, 76.84, and 95.05%, respectively) and restored the depletion of NP-SH and increased PGE2 levels. Moreover, pogostone at a dose of 20 and 40 mg/kg was able to reduce the levels of the proinflammatory cytokines TNF-*α* and IL-6, however, the low dose did not achieve statistical significance. On the other hand, pogostone at all tested doses increased the anti-inflammatory factor IL-10 in a dose-dependent manner. In conclusion, the results indicate a cytoprotective role of pogostone affording gastroprotection against gastric damage induced by ethanol, which is possibly mediated, in part, by endogenous prostaglandins, enhancement of antioxidant activity, and reduction of the secretions of proinflammatory mediators, including the high level of anti-inflammatory cytokine in rats exposed to ethanol.

*β*-Patchoulene (*β*-PAE) and patchoulene epoxide (PAO), obtained from the essential oil of *Pogostemon cablin* (Blanco) Benth., were evaluated concerning the protective effect against ulcers produced by indomethacin and in ethanol-induced gastric ulcer models, respectively. Wua et al. [118] demonstrated that *β*-PAE (10, 20, and 40 mg/kg) reduced gastric damage in the order of 33.84%, 61.53%, and 78.40%, respectively. The histopathological analysis confirmed that *β*-PAE-treated groups displayed less mucosal damage by indomethacin according to the decreasing ulcer area. The authors also showed that *β*-PAE exerted an antiulcer effect by inhibiting TNF-*α*-activated, NF-κB, and JNK signaling pathways. Moreover, Liang et al. [119] evidenced that pretreatment with PAO (10, 20, and 40 mg/kg) was also effective in reducing ethanol-induced gastric injuries by 67.23%, 82.15%, and 84.93%, respectively. Additionally, PAO was able to reverse the increase in the levels of proinflammatory cytokines (TNF-*α* and IL-1*β*) induced by ethanol, also modulating the expression of NF-κB-pathway-related proteins, including p-IκBα, IκBα, p-p65, and p65. Taken together, these results show that *β*-PAE and PAO have a broad spectrum of gastroprotective activity that overcomes the harmful actions of indomethacin or ethanol on the gastric mucosa and reveal the potential use of these compounds as therapeutic agents in the treatment of ethanol- or NSAID-associated gastropathy.

#### 2.1.8. Cell Proliferation

Epidermal growth factor (EGF) is a protein responsible for activating mesenchymal and epithelial cells, stimulating epidermal proliferation and repair after injury [120]. Previous studies have demonstrated that EGF is involved in gastric ulcer healing in experimental gastric and duodenal ulcers in animal models, for example, cold restraint stress [121] and acetic acid [122]. This polypeptide inhibits gastric acid secretion, increases mucus release, and is active in upper and lower gastrointestinal lesions, stimulating cells involved in the healing process [123,124].

The vascular endothelial growth factor (VEGF) participates in the protection of the gastric mucosa, increasing vascular permeability and reducing the area of hemorrhagic lesions [123]. In addition, this peptide can contribute to the development of the angiogenic response that regulates the reconstruction of microvessels and connective tissue cells, contributing restoration of the mucosal architecture, thereby promoting ulcer healing [124,125]. The production of VEGF, occurring in the normal gastric mucosa, is significantly enhanced in the gastric mucosa after injury induced by alcohol [126], dexamethasone [127], or acetic acid [128]. Hence, these growth factors (EGF and VEGF) stimulate with variable power all the cellular elements needed to cure ulcers.

Recently, Bueno et al. [129] investigated the mechanisms of the essential oil from *Baccharis trimera* (EOBT) in gastroprotection against acute gastric ulcer lesions caused by absolute ethanol and a chronic model of gastric lesions induced by acetic acid in rats. This study showed that oral pretreatment with EOBT (100 and 200 mg/kg) decreased gastric injury by 94% and 98%, respectively, in ethanol-induced gastric ulcers. In the gastric lesions induced by acetic acid, EOBT (100 mg/kg) was effective in healing gastric ulcers after 10 (65.5%) and 14 days (61%) of treatment. However, EOBT (100 mg/kg) only once a day for seven days was not able to heal gastric lesions, despite reducing the lesion area (46.7%). To determine whether vascular endothelial growth factor (VEGF) and epidermal growth factor (EGF) are involved in the gastroprotection of EOBT, gastric lesions induced by acetic acid were assessed. The data showed that there was no change in EGF expression in the stomach of rats treated for 14 days with EOBT (100 mg/kg). While in the gastric mucosa of rats treated with the essential oil, there was a significant increase in VEGF expression when compared to the control group. Taken together, these data suggest that the expression of VEGF but not EGF can favor the acceleration of ulcer healing by EOBT determined at the late stages of this healing process. The analysis of the chemical composition of EOBT indicated that it contains carquejyl acetate, ledol, and carquejol as the major components, which may contribute to the biological properties of this essential oil.

**Figure 13:**
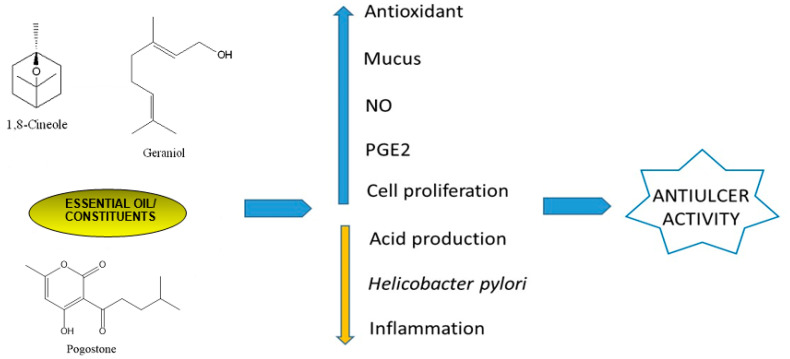
Mechanisms of action proposed for essential oils and constituents with antiulcer activity.

In conclusion, essential oils have been the target of many investigations due to their pharmacological properties, such as the antiulcer effect on models of gastric ulcers. antiulcer effect. The antiulcer activity of these natural products can be attributed to several mechanisms, e.g., antioxidant, inhibition of acid secretion, increase in mucus content, and activity against *H. pylori* (Figure 13).

### 2.2. Mechanisms of Chemopreventive Action of Essential Oils

Cancer is a disease responsible for a high number of deaths annually and is considered a serious public health problem. For example, in 2022, around 20 million new cases of cancer emerged, and 9.7 million deaths caused by this disease were recorded. It is estimated that one in five people of both sexes develops cancer during their lifetime. Also alarming is the proportion of deaths: around one in nine for men and one in twelve for women [130]. The risk factors include family history, tobacco/alcohol use, obesity, infection, and dietary factors, such as low consumption of fruit/vegetables and high consumption of heat-processed meat and red meat. Performing physical activity regularly can also be included as a beneficial recommendation in reducing the occurrence of cancer and other diseases [130,131]. 

In the second half of the last century, more than 600,000 compounds were evaluated for their anticancer activities. However, only about 40 drugs were viable for clinical use [132,133]. There are several challenges to be overcome in searching for new pharmacotherapeutic approaches and chemical agents with anticancer and/or chemopreventive properties. Therefore, the search for new alternative treatments for medicinal and preventive use must be a priority. 

Cancer chemoprevention is established as the use of natural, synthetic, or biological agents to restrict, reverse, or prevent the early stages of carcinogenesis or the advancement of premalignant cells into invasive disease. These agents are known as blockers and suppressors and act by interrupting multiple pathways and processes of tumor development, such as detoxification of electrophilic reactive substances and elimination of free radicals, reduction of cellular uptake, and metabolic activation of procarcinogens, in addition to stimulation of repair pathways [132,134]. 

Many natural agents with chemopreventive properties are found in foods and are present in the diet, including *β*-carotene [135], retinol [132], and lycopene [136], which are not constituents of essential oils. These compounds contain isoprene units in their chemical structures, similar to those found in antitumor monoterpenes from essential oils [137,138]. Therefore, the inclusion of foods rich in these essential oil components in the daily diet could be an interesting strategy to prevent or inhibit the progress of the early stages of cancer. 

Among essential oil components, isoprene derivatives can be highlighted as promising cancer chemopreventive and antitumoral agents [139,140,141,142]. The protective effects of this class of bioactive compounds have been shown in different models of carcinogenesis in rodents [143]. In addition, the inhibitory effects of isoprene derivatives have been extensively shown in several human cancer cell lines [142]. Monoterpenes such as cyclic D-limonene [144] and acyclic geraniol [145,146] and sesquiterpenes such as acyclic farnesol [147] are examples of promising isoprene derivatives for their cancer chemopreventive and antitumoral potential.

Initial cancer chemopreventive and antitumoral mechanistic focus was directed towards isoprene derivatives’ inhibitory effects on 3-hydroxy-3-methylglutaryl coenzyme A (HMG-CoA) reductase activity [140,142]. This is the main enzyme regulating cholesterol biosynthesis in mammalian cells through the mevalonate pathway [148]. It is particularly important for normal cell proliferation as it provides cholesterol, which is a component of cellular membranes, as well as farnesyl and geranylgeranyl pyrophosphates that activate proto-oncogenes including RAS and RHO [148]. This isoprenylation process is key for their binding to the plasma membrane and cell signaling [149]. Different cancer cells including hepatic and mammary ones present higher HMG-CoA reductase activity due to loss of its cholesterol-mediated negative feedback control, favoring cell cycle progression [142,150,151]. Both mammal and plant cells share the initial steps of the mevalonate pathway, which originates cholesterol in the former and the thousands of isoprene derivatives in the latter [152]. Interestingly, while cholesterol transcriptionally inhibits HMG-CoA reductase, essential oils’ isoprene derivatives posttranscriptionally inhibit this enzyme [153,154]. Of note, cancer cells are especially sensitive to isoprene derivatives’ HMGCoA reductase negative feedback regulation, highlighting this enzyme as a key molecular target for cancer chemoprevention and treatment [142]. Importantly, in recent years, other molecular pathways and mechanisms implied in this class of cancer chemopreventive and antitumoral agents have been identified [144,145,147].

D-Limonene is a cyclic monoterpene found in the essential oils of citrus fruit peels including oranges, lemons, limes, and tangerines [155]. Inhibition of proto-oncogene prenylation is one of its main anticancer molecular mechanisms [156,157]. This can be accomplished through posttranscriptional inhibition of HMG-CoA reductase or farnesyl protein transferases [157,158]. Rodent skin carcinogenesis chemoprevention by D-limonene was associated with Ras signaling, as well as anti-inflammatory, antioxidant, and proapoptotic actions through increased Bax and decreased Bcl-2 expression [159]. Accumulating evidence shows that the monoterpene’s main anticancer cellular mechanisms include induction of apoptosis [144,160]. D-Limonene inhibition of lung cancer growth in vitro and in vivo involved induction of apoptosis, which was related to increased levels of cleaved PARP and Bax and decreased levels of Bcl-2, suggesting that the mitochondria-mediated intrinsic death pathway could be involved in cell death induction [161]. In addition, in this study, D-limonene autophagy induction, which was accompanied by increased atg5 expression, could be relevant for the apoptosis effects [161]. Increased Bax and caspase-3 and decreased Bcl-2 expression were associated with G2/M arrest and apoptosis induction in T24 bladder cancer cells [162]. A D-limonene-rich blood orange (*Citrus sinensis* (L) Osbeck) volatile oil was shown to dose-dependently inhibit cell proliferation and induce apoptosis, as well as to inhibit angiogenesis in SW480 and HT-29 human colon cancer cells [163]. These effects involved dose-dependent induction of Bax/Bcl-2 and inhibition of vascular endothelial growth factor (VEGF) expression [163]. Suppression of the PI3K/Akt pathway was suggested to be related to D-limonene apoptosis induction in LS174T human colon cancer cells [164]. Furthermore, MAP38 and ERK pathways are relevant ways of mediating D-limonene apoptosis induction in BW5147 murine lymphoma cells [165].

Geraniol is an acyclic monoterpene found in the essential oils of several aromatic plants such as lavender, citronella, and lemongrass [166,167]. It presented chemopreventive activities in rodent models of mammary [168], liver [152,169], renal [146], and tongue [170] carcinogenesis. In addition, it presented inhibitory effects in several cancer cell lines [145,146]. Geraniol’s cancer protective effects involve HMG-CoA-reductase-related [168,171] or independent effects [152,172]. In addition, geraniol has been shown to modulate oxidative stress [170], inflammation [173], cell proliferation [174], and apoptosis [152]. In vivo chemopreventive activities against tongue carcinogenesis by geraniol involved inhibition of phase I enzymes and induction of phase II antioxidant and carcinogen detoxifying enzymes via activation of transcription factor Nrf-2 [170]. In addition, apoptosis induction by geraniol during rodent carcinogenesis was accompanied by modulation of different molecular targets including Kim-1, NF-κB, PCNA, and p53 [146]. In vitro and in vivo growth inhibitory effects by geraniol against A549 human lung adenocarcinoma cells involved inhibition of cell proliferation and induction of apoptosis [175]. These protective effects of geraniol involved decreased activity of HMG-CoA reductase that was accompanied by decreased Ras binding to the plasma membrane [175]. On the other hand, hepatocarcinogenesis chemoprevention by geraniol involved inhibition of RhoA binding to the plasma membrane without decreasing HMG-CoA reductase, suggesting that the monoterpene could have inhibited the oncogene prenylation through geranylgeranyl transferase inhibition [169]. In MCF-7 human breast cancer cells, geraniol treatment elicited G1 and G2/M growth arrest and cell proliferation inhibition [172]. These effects were accompanied by inhibition of CDK 2 activity and expression of cyclins D1, E, and A, and CDKs 2 and 4, although inhibition of the mevalonate pathway through HMG-CoA reductase inhibition did not exert a relevant role [172]. Interestingly, inhibition of Hep-2 human hepatic cancer cell proliferation by geraniol involved inhibition of the mevalonate pathway at a step after lanosterol synthesis, suggesting that the monoterpene effects on cholesterol synthesis can be independent of HMG-CoA reductase modulation [174]. In addition, geraniol’s antiproliferative effects were accompanied by inhibition of protein prenylation [174]. Other relevant geraniol molecular targets in cancer cells include E2F8A [176] and HSP90 [177]. A systematic computational-based approach via network pharmacology proposed 38 potential geraniol molecular targets, several of which are involved in cancer pathways (estrogen receptors, G protein subunits, caspases, MAP kinases, ornithine decarboxylase 1, among others) [178]. This reinforces geraniol as a multi-targeted anticancer agent that seems to act through several molecular pathways, a feature that increases its effectiveness for cancer control [179]. Of note, geraniol was recently shown to upregulate PTEN through the downregulation of mir-21 in an in vivo breast cancer model [180]. This suggests that the monoterpene could also modulate epigenetic processes that are key for cancer prevention.

Farnesol is an acyclic sesquiterpene found in the essential oils of several aromatic plants such as lemongrass, citronella, rose, and musk [147]. It has presented chemopreventive activities in rodent models of pancreatic [181], liver [141,182], colon [183], and skin [184] carcinogenesis. In addition, it presented inhibitory effects in several cancer cell lines [147,185]. Farnesol’s cancer protective effects involve HMGCoA-reductase-related [152,186] or independent effects [183]. In addition, farnesol has been shown to modulate oxidative stress [187], inflammation [182], cell proliferation [152], and apoptosis [188]. Farnesol’s protective effects against chemical hepatocarcinogenesis were associated with the induction of SOD, GPX, and CAT antioxidant enzymes as well as inhibition of COX-2 and TNF-α proinflammatory markers [182]. Similar effects on these antioxidant enzymes were reported after farnesol treatment in rats submitted to colon [189] and lung [190] carcinogenesis. Skin carcinogenesis prevention by sesquiterpene involves inhibition of oxidative stress and inflammation, as well as reduction of cell proliferation and induction of apoptosis [184]. These protective effects were related to the inhibition of COX-2 and of the Ras/Raf/p-ERK1/2 pathway and to an increase in the proportion of Bax/Bcl-2. These results indicate that farnesol acts by interfering with multiple signaling pathways [184]. Apoptosis induction by farnesol in DU145 human prostate cancer cells involved PI3K/Akt and mitogen-activated protein kinase signaling pathways [191]. Similarly, HeLa human cervical cancer cells treated with farnesol also exhibited increased apoptosis and downregulation of the PI3K/Akt pathway [192]. Furthermore, other molecular targets have been implied in farnesol’s proapoptotic effects in cancer cells including PPARγ [193], STAT-3 [194], and the TF4-ATF3-CHOP cascade of ER stress [195]. 

### 2.3. Mechanisms of Cardiovascular Action of Essential Oils

Cardiovascular diseases (CVDs) have a major impact on global health as shown in the World Health Organization (WHO) reports, accounting for 31% of total deaths worldwide. It is expected that by 2030, 20% of the world’s population over 65 will have 40% of CVD deaths. Hypertension is more prevalent in developed countries, affecting approximately 45% of the general population, due to incorrect lifestyle and behavioral habits such as diets, alcohol abuse, physical inactivity, and stress [196]. Hypertension is characterized by a blood pressure greater than 130/80 mm Hg, according to the American College of Cardiology (ACC)/American Heart Association (AHA) guidelines and is strongly correlated with a high incidence of mortality, as it is the most direct causal risk factor for cardiovascular disease (CVD) leading to stroke and ischemic heart disease [197]. 

Anantha et al. [198] emphasized that blood pressure is a result of cardiac output and systemic vascular resistance. Vascular tone may be elevated due to increased α-adrenoceptor stimulation or increased peptides such as angiotensins or endothelins. The renin–angiotensin system is involved at least in some forms of hypertension (e.g., renovascular hypertension) and is suppressed in the presence of primary hyperaldosteronism. In this context, Takimoto-Ohnishi and Murakami [199] reported that the renin–angiotensin system (RAS) is a regulatory cascade that plays major physiological roles in blood pressure regulation and electrolyte homeostasis. The homeostasis of body fluids and sodium is controlled by the renin–angiotensin system. Angiontensin II is the most potent hormone of this system, as it plays an important role in regulating vascular tone, cardiac function, and renal sodium reabsorption [200].

**Figure 14:**
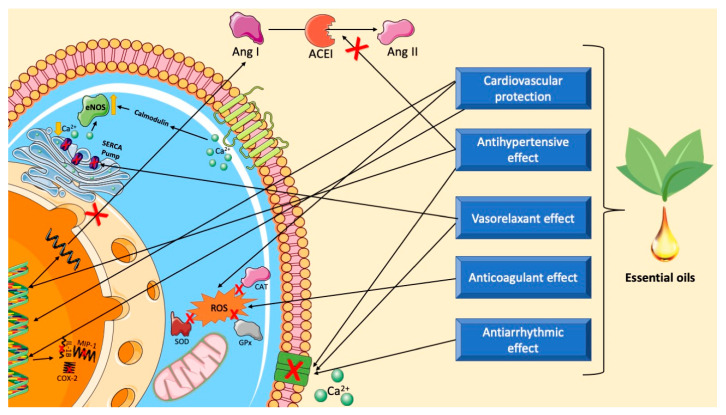
Pharmacological effects and potential targets of essential oils from plants against systemic arterial hypertension and implications thereof. Legend: Ang I—Angiotensin I; ACEI—Angiotensin I-converting enzyme inhibitor; Ang II—Angiotensin II; eNOS—endothelial nitric oxide synthase; SERCA—sarcoplasmic/endoplasmic reticulum Ca^2+^—ATPase; MIP-1—macrophage inflammatory protein-1; COX-2—cycloxigenase-2; IL-1β—interleukin-1β; CAT—catalase; ROS—reactive oxygen species; SOD—superoxide dismutase; GPx—glutathione peroxidase.

Currently, there are several groups of drugs widely used in the treatment of hypertension, such as diuretics, beta-blockers, angiotensin-converting enzyme (ACE) inhibitors, Ca^2+^ channel blockers, angiotensin II receptor antagonists, and renin inhibitors. However, some of the substances in these drug groups have significant side effects, mainly on the central nervous system [201]. As a result, studies indicate the effectiveness of compounds present in essential oils in reducing blood pressure and heart rate [202]. Figure 14 and Table 1 summarize some mechanisms underlying antihypertensive effects of essential oils.

#### 2.3.1. Inhibition of Angiotensin Converting Enzyme (ACE)

Suručić et al. [203] described that *Seseli pallasii* Besser (Apiaceae) essential oil relaxed isolated endothelium intact mesenteric arteries’ rings precontracted with phenylephrine with IC_50_ = 3.10 nl/mL (IC_50_ = 2.70 μg/mL). The *S. pallasii* essential oil was found to exhibit a dose-dependent ACE inhibitory activity with an IC_50_ value of 0.33 mg/mL. The results suggested that a combination of vasorelaxing and ACE inhibitory effects of the *S. pallasii* essential oil might have potential therapeutic significance in hypertension.

Adefegha and Oboh [204] compared the action of captopril and essential oils of the Zingiberaceae family in hypertensive rats, and both inhibited angiotensin I converting enzyme activity, with essential oils having the highest ACE inhibitory activity, and this was attributed to the major constituent from *Aframomum melegueta* K. Schum. (alligator pepper) and *Aframomum daniellii* K. Schum. (African or “false” cardamom), eugenol.

The essential oil from *Blepharocalyx salicifolius* (Kunth) O. Berg (Myrtaceae family) showed 34 compounds. The sesquiterpenes were the largest fraction of 55.9%. The main component was spathulenol with 11.6%, which caused an antihypertensive effect with a decrease in systolic blood pressure (SBP) and diastolic blood pressure (DBP) (12% and 23%, respectively) and a decrease in cardiac ACE activity of 41.5% in spontaneously hypertensive rats [212].

#### 2.3.2. Modulation NO/cGMP Pathway

The essential oil from *Pogostemon elsholtzioides* Benth. (Lamiaceae) induced dose-dependent vasodilation in precontracted aortic rings and a vasorelaxant effect, attributed to the major constituents, such as curzerene (46.10%), benzophenone, α-cadinol, and germacrone. There was also an antihypertensive effect, observed in hypertensive rats that had a systolic pressure of 100 mmHg and a diastolic pressure of 64.77 mmHg at the beginning of the experiment. After administration of EO, there was a decrease to 84.18 mmHg of systolic pressure and 21.69 mmHg of diastolic pressure. The vasorelaxant effect in rat aortic rings involves the activation of both nitric oxide synthase and K^+^ channels, which indicates the possible role of the NO/cGMP/PKG pathway in this effect [205].

*Alpinia zerumbet* has demonstrated antihypertensive and vasodilator effects. Rocha et al. [206] pointed out that the essential oils of *Alpinia zerumbet* showed an endothelium-independent vasorelaxant effect in second-order branches of the mesenteric artery. There was a vasodilator effect mediated by the inhibition of Ca^2+^ influx and release from intracellular storage, as well as an activation of the NOS/sGC pathway, being significant for the treatment of hypertension.

#### 2.3.3. Modulation of Ion Channels

Another interesting experiment showed that the essential oils of *Trachyspermum ammi* (Linn.) Sprague (Apiaceae) seeds caused a completely endothelium-independent vasorelaxation effect and extracellular Ca^2+^ influx, attributed to the main constituents: thymol (38.1%), gamma-terpinene (33.3%), and *p*-cymene (23.1%) [207]. Jamhiri et al. [213] stated that thymol has a strong antioxidant potential that causes cardioprotective effects and prevents left ventricular hypertrophy by increasing serum antioxidant capacity.

Dib et al. [208] showed that *Artemisia campestris* L. (Asteraceae) essential oil (AcEO) had a vasorelaxation effect, acting through L-type calcium channels and SERCA pumps to reduce intracellular calcium, and, consequently, triggers sustained vasodilation. The maximal antioxidant effect was obtained with the dose of 2 mg/mL of AcEO. A dose of 1 mg/mL showed a maximum antiplatelet effect of 49.73% ± 9.54 and 48.20% ± 8.49, respectively, on thrombin and ADP. The main components of this oil are spathulenol, *β*-eudesmol, and *p*-cymene. 

The essential oil from *Lippia alba* (Mill.) N.E. Brown (Verbenaceae), known as lemon balm, is a strong vasorelaxant in the isolated aorta, probably due to the presence of the citral compound that causes blockage of Ca^2+^ influx across the cell membrane or changes in calcium binding protein sensitization and/or intracellular calcium storage [209]. 

The cumulative addition of essential oil nanoemulsion (EONE) of *Chrysopogon zizanioides* (L.) Roberty (Poaceae) produced a vasorelaxant effect in thoracic aortic rings of spontaneously hypertensive rats. This essential oil possesses a vasorelaxant effect through the muscarinic pathway as well as acts as a calcium channel blocker. The study shows that *C. zizanioides* root EO has the potential for further investigation as a possible antihypertensive drug [210]. 

2-Phenyl-ethyl alcohol, an isolated component of *Rosa damascena* Mill (Rosaceae) essential oil, exhibited a potent vasorelaxation effect in the mesenteric artery and aorta of hypertensive rats. The activation of calcium-sensitive potassium channels might be the putative mechanism of the vasorelaxant effect [211]. 

### 2.4. Antidiabetes Action Mechanisms of Essential Oil

Diabetes mellitus is a chronic disease that affects around 3% of the worldwide population, with a prospect of increasing by 2030, and its prevalence has increased given the aging population. In 2015, the International Diabetes Federation (IDF) estimated that 1 in 11 adults aged 20 to 79 years had type 2 diabetes mellitus. Diabetes mellitus ranks ninth among diseases that cause loss of healthy life years [214]. About 422 million people have DM and 1.6 million deaths are directly attributed to the disease each year [215]. Likewise, an increase in the number of deaths related to DM between 1990 and 2019, from 1,278,866 to 2,988,924, has been reported. For disability-adjusted life years (DALYs), growth was observed from 28,586,671 in 1990 to 70,888,154 in 2019. 

Diabetes mellitus (DM) is a metabolic disorder of multiple etiologies, which is mainly characterized by persistent elevation of the level of glucose in the blood (hyperglycemia), with disturbances in the metabolism of carbohydrates, lipids, and proteins, resulting from a deficiency in insulin secretion, its action, or both [216,217,218]. 

**Figure 15:**
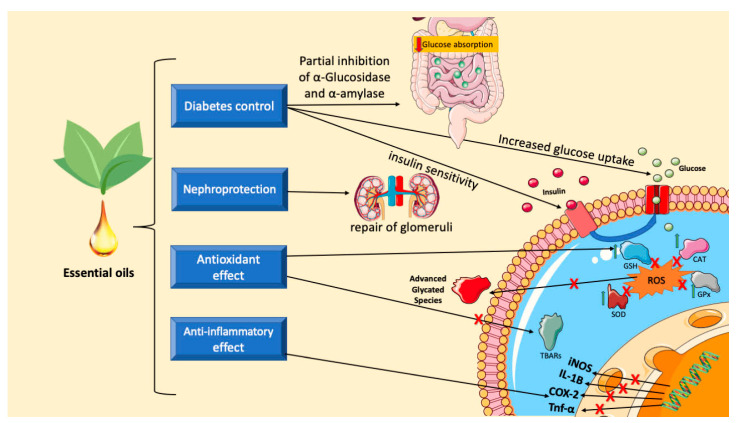
Pharmacological effects and potential targets of essential oils from plants against diabetes mellitus. Legend: iNOS—inducible nitric oxide synthase; COX-2—cycloxigenase-2; IL-1*β*—interleukin-1*β*; TNF-*α*—tumor necrosis factor-*α*; CAT—catalase; ROS—reactive oxygen species; SOD—superoxide dismutase; GSH—reduced gluthatione; GPx—glutathione peroxidase; TBARS—thiobarbituric acid reactive substances.

Essential oils have a complex chemical composition that allows them to reach multiple physiological structures and metabolic processes of antidiabetic aspects (see Figure 15 and Table 2), with several mechanisms of action described [219]. Therefore, these products and their components may have antiglycemic actions and potential use as pharmacologically active chemical agents capable of reducing the harmful effects of diabetes mellitus on the health of people affected by the disease. Additional investigations must be carried out, including clinical studies, to verify the viability of these compounds as candidates for antidiabetic drugs.

#### 2.4.1. Inhibition of *α*-Amylase and *α*-Glucosidase

Oboh et al. [220] investigated *Citrus sinensis* L. Osbeck (orange) and *Citrus limon* (L.) Burm. f. (lemon) essential oils and their interactions with *α*-amylase, *α*-glucosidase, and ACE activities. The results showed that the EO inhibited *α*-amylase and *α*-glucosidase activities. The components of EO from orange and lemon peels contained mostly monoterpene hydrocarbons, oxygenated monoterpenes, and sesquiterpenes. Limonene was the main constituent, 92.14% in orange and 53.07% in lemon. In a study carried out with D-limonene administered to streptozotocin-induced diabetic rats, it was demonstrated that this monoterpene alters several biochemical parameters associated with diabetes and, therefore, can contribute to preventing clinical complications arising from this disease [230]. In this way, the antienzymatic actions of *Citrus sinensis* L. Osbeck and *Citrus limon* (L.) Burm. f. essential oils must be related to the antidiabetic activity of their majority constituent, limonene. 

A study by Lekshmi et al. [231] showed that turmeric essential oil (*Curcuma longa* L.—Zingiberaceae) has a protective effect against type 2 diabetes through different mechanisms involving a hypoglycemic effect attributed to upregulation of insulin sensitivity and lower cellular glucose uptake. The major constituent of *Curcuma longa* EO is *ar*-turmerone, which can inhibit the activities of *α*-amylase (IC_50_ = 24.5 μg/mL) and *α*-glucosidase (IC_50_ = 0.28 μg/mL), two key enzymes in glucose digestion. This biological activity was also evidenced in *Mentha viridis* Linn (Lamiaceae) essential oil, which showed a significant ability to inhibit α-amylase (IC_50_ = 101.72 μg/mL) and *α*-glucosidase (IC_50_ = 86.93 μg/mL) [232].

In a profile study of 62 essential oils from different plant species and botanical families, hypoglycemic activity was evidenced in an enzymatic assay of *α*-amylase and chemical characterization. The different plants belonged to the following families*:* Lauraceae, Myristicaceae, Myrtaceae, Oleaceae, Pinaceae, Piperaceae, Poaceae, Rosaceae, Rutaceae, Santalaceae, Verbenaceae, and Zingiberacea. In vitro *α*-amylase inhibitory potentials were obtained after evaluation of *Eucalyptus radiata* A. Cunn, *Laurus nobilis* L. (Lauraceae), and *Myristica fragrans* Houtt. (Myristicaceae) EOs. These products exhibited inhibitory capacities comparable to those of the positive control (acarbose) [222].

In the study conducted by Radünz et al. [221], among all the EOs evaluated, thyme EO had the most significant inhibition of *α*-glucosidase (98.9%), followed by sweet orange EO (*Citrus sinensis* L. Osbeck, Rutaceae) which showed the most potent inhibitory effect on *α*-amylase (95.4%). The inhibitory effects of thyme and orange EOs were attributed to their main components, thymol and D-limonene, respectively. Incomplete enzyme inhibition and medium- and high-range inhibition for *α*-amylase and *α*-glucosidase, respectively, are interesting for clinical treatment as they allow diabetes control without compromising nutrients or glucose absorption in the small intestine.

Nait-Irahal et al. [223] analyzed the effect of intraperitoneal administration of clove essential oil (*Syzygirum aromaticum* L., Myrtaceae) in diabetic rats. A significant reduction in blood glucose, total cholesterol, and xanthine oxidase levels and potent inhibition of the α-amylase enzyme were observed. In another study, the essential oil from *Ocimum basilicum* L. (Lamiaceae) demonstrated strong α-amylase inhibitory activity (IC_50_ = 74.13 μg/mL). The essential oil is mainly composed of linalool, methyl estragole, methyl cinnamate, and methyl chavicol [224]. Linalool has many pharmacological actions. There are even reports of the nephroprotective action of this monoterpene in diabetic rats [233]. These data highlight the therapeutic potential of this natural product for the treatment of diabetes. 

*Serevenia buxifolia* (Poir.) Ten. (Rutaceae) is a perennial citrus plant whose essential oil has antidiabetic potential, with IC_50_ values of 87.8 and 134.9 μg/mL against *α*-amylase and *α*-glucosidase, respectively. The biological action was concentration-dependent. This oil contains 33 components, such as *β*-caryophyllene (32.5%) and elixene (9.8%) [225]. There are several reports of the antidiabetic action of *β*-caryophyllene. This sesquiterpene was administered orally in a dose-dependent manner to diabetic rats, in combination with the hypoglycemic drug glibenclamide (600 μg/kg body weight), for 45 days. There was a significant reduction in glucose due to the increase in plasma insulin levels and the actions of carbohydrate metabolic enzymes were almost normalized [234]. In another study, this sesquiterpene had an insulinotropic and antidiabetic action when combined with L-arginine. The experiments were carried out in type 2 diabetic rats and a decrease in glucose was observed. Additionally, there was a reduction in lipid levels and oxidative stress [235]. So, scientific reports demonstrate the high potential of this natural product for restoring glucose homeostasis.

*Kaempferia galanga* (L.) is a plant with aromatic rhizomes and belongs to the Zingiberaceae family. Its essential oil has a high concentration of ethyl *p*-methoxycinnamate and shows *α*-amylase inhibitory activity with an IC_50_ value of 18.50 μg/mL. Acarbose, an antidiabetic drug, had IC_50_ = 20.39 μg/mL [226]. Chander et al. [227] also found excellent in vitro action of *Dracocephalum heterophyllum* Benth. essential oil (Lamiaceae) in inhibiting the enzymes *α*-amylase and *α*-glucosidase. *β*-Citronellol (31.5–83.7%) is the major constituent in samples of this oil. The antidiabetic action of citronellol has been demonstrated in an experimental model using streptozotocin-induced diabetic rats. This monoterpene was administered to animals orally (50 mg/kg) for 30 days and attenuated hyperglycemia via the action of strategic carbohydrate metabolic enzymes [236].

The essential oils of the Moroccan medicinal plants *Mentha suaveolens* “Variegata” (Labiatae), *Lavandula stoechas* L. (Lamiaceae), and *Ammi visnaga* (L.) Lam. (Apiaceae) were evaluated for their in vitro ability to inhibit the enzymes *α*-amylase and *α*-glucosidase. The study by El Hachlafi et al. [228] highlighted the outstanding biological action of the three plant species, whose essential oils were able to repress the activities of the enzymes in low concentrations compared to the standard drug used (acarbose). *Mentha suaveolens* essential oil mainly contains the constituents fenchone (29.77%) and camphor (24.90%). These monoterpenes are also the main components of *Lavandula stoechas* L. essential oil (fenchone (31.81%) and camphor (29.60%)). The three products were tested against the enzymes *α*-amylase and *α*-glucosidase. They showed inhibitory activity with IC_50_ of 106.73, 104.19, and 76.92 μg/mL against *α*-amylase and 98.54, 69.03, and 105.26 μg/mL in assays using α-glucosidase [237]. Therefore, these data indicate that fenchone and camphor should contribute to the antidiabetic action of *Mentha suaveolens* essential oil.

#### 2.4.2. Oxidative Stress and Inflammation-Related Mechanisms

The essential oil from *Hypericum scabrum* L. (Clusiaceae) has a protective and healing effect on wounds in streptozotocin-induced diabetic rats. The administration of *Hypericum scabrum* L. essential oil caused an increase in the level of GSH, GPx, SOD, and CAT activities and there was a decrease in the levels of MDA. *H. scabrum* L. essential oil may help reduce lipid peroxidation, oxidative stress, and the associated complications that go along with them, in addition to playing a beneficial role in the management of diabetic wounds [229]. Gandhi et al. [238] discussed in a data analysis that constituents of essential oils, including linalool, cinnamaldehyde, zerumbone, myrtenol, thujone, *α*-terpineol, geraniol, citral, eugenol, thymol, carvacrol, citronellol, and thymoquinone, have antidiabetic properties through modulation of metabolic pathways associated with glucose metabolism. For example, these constituents of essential oils can reduce the expression of TNF-*α*, IL-4, iNOS, and COX-2, as well as regulate signaling molecules, such as AMPK, caspase-3, NF-κB, and Nrf2/ HO-1.

## 3. Conclusions

The synthetic routes presented to obtain bioactive derivatives demonstrate the importance of the constituents of these oils as starting materials in the synthesis of strategic products for several sectors, particularly for the pharmaceutical sector. The interference of these oils in various metabolic pathways, inhibiting or activating metabolites related to organic disorders associated with diseases such as diabetes, hypertension, cancer, and ulcers, suggests the promising use of these products as prototypes for new drugs. Additional studies are necessary to advance the understanding of the pharmacological activities and toxicological aspects aiming to establish the therapeutic potential of these components and their synthetic derivatives in a clinical stage.

## Figures and Tables

**Table 1:** Essential oils from plant species and their pharmacological mechanisms on hypertension.

Plant Species (Family)	Major Compounds from Essential Oils	Pharmacological Action	References
*Seseli pallasii* Besser (Apiaceae)	*α*-Pinene (42.7–48.2%)	Vasorelaxant and ACE-inhibiting effects	[203]
*Aframomum melegueta* (Roscoe) K. Schum. and *Aframomum daniellii* (Hook.f.) K. Schum. (Zingiberaceae)	Eugenol *A. melegueta*: 82.2% *A. daniellii*: 51.1%	EO inhibited angiotensin I-converting enzyme activity	[204]
*Pogostemon elsholtzioides* Benth. (Lamiaceae)	Curzerene: 46.1%	Involvement of nitric oxide synthase and K^+^ channel activation	[205]
*Alpinia zerumbet* (Pers.) B.L.Burtt & R.M.Sm. (Zingiberaceae)	1,8-Cineole (24.2%), terpinen-4-ol (20.4%), and *p*-cymene (15.7%)	Vasodilator effect mediated by inhibition of Ca^2+^ influx and release from intracellular storage, as well as an activation of the NOS/sGC pathway	[206]
*Trachyspermum ammi* Sprague (Apiaceae)	Thymol (38.1%), gamma-terpinene (33.3%), and *p*-cymene (23.1%)	Vasorelaxant effect by inhibition of extracellular Ca^2+^ influx via calcium channels	[207]
*Artemisia campestris* L. (Asteraceae)	Spathulenol: 10.1%	Vasorelaxation induced by AcEO via L-type calcium channels	[208]
*Lippia alba* (Mill.) N.E.Br. (Verbenaceae)	Citral	Vasorelaxant effect in isolated aorta, via three hypothesized mechanisms: blockade of Ca^2+^ influx or changes in calcium binding protein sensitization and/or intracellular calcium storage	[209]
*Chrysopogon zizanioides* (L.) Roberty (Poaceae)	Khusimol (8.2%), *β*-vetivenene (8.2%), *β*-funebrene (5.1%), *β*-vetispirene (4.8%), *β*-vetivone (4.7%), *δ*-selinene (4%), (*E*)-isovalencenol (3.3%), *α*-vetivone (3.3%), *β*-calacorene (3%), vetivonic acid (2.9%), and vetiselinenol (2.8%).	The root essential oil of *C. zizanioides* possesses a vasorelaxant effect through the muscarinic pathway as well as acts as a calcium channel blocker	[210]
*Rosa damascena* Mill. (Rosaceae)	2-Phenyl-ethyl	Vasorelaxation by activation of large-conductance Ca^2+^-activated K^+^ (BKCa) channels	[211]

**Table 2:** Essential oils from plant species and their pharmacological mechanisms on diabetes.

Plant Species (Family)	Major Compounds from Essential Oils	Pharmacological Action	References
*Citrus × sinensis* (L.) Osbeck (Rutaceae) and *Citrus limon* (L.) Burm. f. (Rutaceae)	D-Limonene *C. sinensis*: 92.14% *C. limon*: 53.07%	The EO inhibited *α*-amylase and *α*-glucosidase activities	[220,221]
62 species from the families: Lauraceae, Myristicaceae, Myrtaceae, Oleaceae, Pinaceae, Piperaceae, Poaceae, Rosaceae, Rutaceae, Santalaceae, Verbenaceae, and Zingiberaceae	-	In vitro α-amylase inhibitory potentials were obtained after the evaluation of *Eucalyptus radiata*, *Laurus nobilis,* and *Myristica fragrans* EOs.	[222]
*Syzygirum aromaticum* L. (Myrtaceae)	Eugenol	There was a significant decline in blood glucose levels, total cholesterol, xanthine oxidase, antioxidant activities, and it was a potent *α*-amylase inhibitor	[223]
*Ocimum basilicum* L. (Lamiaceae)	Linalool, methyl estragole, methyl cinnamate, and methyl chavicol	*Ocimum basilicum* essential oil had a strong *α*-amylase inhibitory activity	[224]
*Serevenia buxifolia* (Poir.) Ten. (Rutaceae)	*β*-caryophyllene (32.5%) and elixene (9.8%)	Antidiabetic potential by inhibiting the enzymes *α*-amylase and *α*-glucosidase	[225]
*Kaempferia galanga* (L.) (Zingiberaceae)	Ethyl *p*-methoxycinnamate: 66.39%	Antidiabetic activity using α-amylase inhibitory activity assay	[226]
*Dracocephalum heterophyllum* Benth. (Lamiaceae)	-	Antidiabetic potential by inhibiting the enzymes *α*-amylase and *α*-glucosidase	[227]
*Mentha suaveolens* “Variegata” (Labiatae) (MSEO); *Lavandula stoechas* L. (Lamiaceae) (LSEO); *Ammi visnaga* (L.) Lam. (Apiaceae) (AVEO)	MSEO: fenchone (29.77%) and camphor (24.90%) LSEO: piperitenone oxide (74.55%) AVEO: linalool (38.24%)	Antidiabetic action by inhibiting the enzymes *α*-amylase and *α*-glucosidase	[228]
*Hypericum scabrum* L. (Clusiaceae)	-	The administration of *Hypericum scabrum* L. essential oil caused an increase in the level of GSH, GPx, SOD, and CAT activities and there was a decrease in the levels of MDA	[229]

## Abbreviations

CAT	Catalase
cGMP	Cyclic guanosine monophosphate
COX-1	Cyclooxygenase 1
COX-2	Cyclooxygenase 2
DPPH	2,2-Diphenyl-1-picrylhydrazyl
GSH	Glutathione
H^+^/K^+^ ATPase	Hydrogen potassium ATPase
IL-1*β*	Interleukin 1*β*
IL-6	Interleukin 6
IL-10	Interleukin 10
L-NAME	N (G)-nitro-L-arginine methyl ester
MDA	Malondialdehyde
MIC	Minimum inhibitory concentration
MPO	Myeloperoxidase
NF-κB	Nuclear factor kappa B
NO	Nitric oxide
NOS	Nitric oxide synthase
NP-SH	Non-protein Sulfhydryl
NSAIDs	Non-steroidal anti-inflammatory drugs
PGE2	Prostaglandin E2
p-IκBα and IκBα	Inhibitors of the transcription factor NF-κB
p-p65 and p65	NF-κB subunits
ROS	Reactive oxygen species
SOD	Superoxide dismutase
TNF-*α*	Tumor necrosis factor alpha
